# The evidence-based role of catecholaminergic PET tracers in Neuroblastoma. A systematic review and a head-to-head comparison with mIBG scintigraphy

**DOI:** 10.1007/s00259-023-06486-9

**Published:** 2023-11-14

**Authors:** Arnoldo Piccardo, Giorgio Treglia, Francesco Fiz, Zvi Bar-Sever, Gianluca Bottoni, Lorenzo Biassoni, Lise Borgwardt, Bart de Keizer, Nina Jehanno, Egesta Lopci, Lars Kurch, Michela Massollo, Helen Nadel, Isabel Roca Bielsa, Barry Shulkin, Reza Vali, Diego De Palma, Diego Cecchin, Ana Isabel Santos, Pietro Zucchetta

**Affiliations:** 1 Department of Nuclear Medicine, E.O. “Ospedali Galliera”, Mura Delle Cappuccine 14, 16128 Genoa, Italy; 2 Clinic of Nuclear Medicine, Imaging Institute of Southern Switzerland, Ente Ospedaliero Cantonale, Bellinzona, Switzerland; 3 Faculty of Biology and Medicine, University of Lausanne, Lausanne, Switzerland; 4 Faculty of Biomedical Sciences, Università Della Svizzera Italiana, Lugano, Switzerland; 5 Department of Nuclear Medicine and Clinical Molecular Imaging, University Hospital, Tübingen, Germany; 6 Department of Nuclear Medicine, Schneider Children’s Medical Center, Tel Aviv University, Tel Aviv, Israel; 7 Great Ormond Street Hospital for Children, NHS Foundation Trust, London, UK; 8 Rigshospitalet, University of Copenhagen, Copenhagen, Denmark; 9 Department of Nuclear Medicine and Radiology, University Medical Center Utrecht, Utrecht, the Netherlands; 10 Department of Nuclear Medicine, Institut Curie Paris, Paris, France; 11 Nuclear Medicine Unit, IRCCS-Humanitas Research Hospital, Rozzano, Milano Italy; 12 Department of Nuclear Medicine, University Hospital Leipzig, Leipzig, Germany; 13 Department of Pediatric Nuclear Medicine, Lucile Packard Children’s Hospital of Stanford (CA), Palo Alto, USA; 14 Universitat Autònoma de Barcelona, Barcelona, Spain; 15 St Jude Children’s Research Hospital, Memphis, TN USA; 16 Division of Nuclear Medicine, Department of Diagnostic Imaging, The Hospital for Sick Children of Toronto, Toronto, Canada; 17 Nuclear Medicine Unit, Ospedale Di Circolo of Varese, Varese, Italy; 18 Nuclear Medicine Unit, Department of Medicine - DIMED, University Hospital of Padova, Padua, Italy; 19 Department of Nuclear Medicine, Hospital Garcia de Orta, Almada, Portugal

**Keywords:** Guideline, PET-CT, Paediatric PET, Catecholamine, Neuroblastoma, [^18^F]F-DOPA, [^124^I]MIBG, 18F-MFBG, 11C-HED

## Abstract

**Background:**

Molecular imaging is pivotal in staging and response assessment of children with neuroblastoma (NB). [^123^I]-metaiodobenzylguanidine (mIBG) is the standard imaging method; however, it is characterised by low spatial resolution, time-consuming acquisition procedures and difficult interpretation. Many PET catecholaminergic radiotracers have been proposed as a replacement for [^123^I]-mIBG, however they have not yet made it into clinical practice. We aimed to review the available literature comparing head-to-head [^123^I]-mIBG with the most common PET catecholaminergic radiopharmaceuticals.

**Methods:**

We searched the PubMed database for studies performing a head-to-head comparison between [^123^I]-mIBG and PET radiopharmaceuticals including meta-hydroxyephedrine ([^11^C]C-HED), ^18^F-18F-3,4-dihydroxyphenylalanine ([^18^F]DOPA) [^124^I]mIBG and *Meta*-[18F]fluorobenzylguanidine ([^18^F]mFBG). Review articles, preclinical studies, small case series (< 5 subjects), case reports, and articles not in English were excluded. From each study, the following characteristics were extracted: bibliographic information, technical parameters, and the sensitivity of the procedure according to a patient-based analysis (PBA) and a lesion-based analysis (LBA).

**Results:**

Ten studies were selected: two regarding [^11^C]C-HED, four [^18^F]DOPA, one [^124^I]mIBG, and three [^18^F]mFBG. These studies included 181 patients (range 5–46). For the PBA, the superiority of the PET method was reported in two out of ten studies (both using [^18^F]DOPA). For LBA, PET detected significantly more lesions than scintigraphy in seven out of ten studies.

**Conclusions:**

PET/CT using catecholaminergic tracers shows superior diagnostic performance than mIBG scintigraphy. However, it is still unknown if such superiority can influence clinical decision-making. Nonetheless, the PET examination appears promising for clinical practice as it offers faster image acquisition, less need for sedation, and a single-day examination.

## Background

Neuroblastoma (NB) is an embryonic tumour deriving from the sympathetic nervous system able to produce an excess of catecholamines. It is the most common extra-cranial tumour of the paediatric age. In high-risk disease, metastatic lesions are often present at diagnosis, giving a poor prognosis with long-term survival of about 40–50% [1, 2]. In this challenging clinical scenario, intensive and multi-step therapeutic regimens have been developed (i.e., induction chemotherapy, surgery, second-line high-dose chemotherapy, autologous stem cell transplantation, radiation therapy, radionuclide therapy, differentiation therapies, and immunotherapy) to achieve the best treatment for each NB patient [3]. This approach relies on obtaining an accurate diagnostic disease assessment before proceeding to each subsequent therapeutic step. Therefore, effective and reliable diagnostic procedures are in high demand.

Molecular imaging plays a pivotal diagnostic role in staging and evaluating treatment response in patients with NB. [^123^I]-metaiodobenzylguanidine (mIBG) whole-body scintigraphy in combination with SPECT/CT of the chest and abdomen is recognised as the cornerstone imaging procedure to stage and restage NB patients properly [4]. The main advantages over the other conventional imaging procedures (i.e., CT and MRI) are its high diagnostic accuracy in detecting distant metastases and its ability to provide reliable predictive and prognostic information by applying a dedicated scoring system validated over the years [5–7]. In addition, a positive ^123^I-mIBG scan paves the way for using radionuclide therapy with ^131^I-mIBG.

However, the mIBG scan has some diagnostic and practical limitations. Notably, false negative results may occur in about 10% of patients [8, 9]. Furthermore, the spatial resolution of this imaging technique even when combined with SPECT/CT, is suboptimal compared to other cross-sectional imaging tools. Additionally, the mIBG scan requires adequate thyroid blockade. Moreover, it implies at least a two-day protocol, including time-consuming planar and SPECT image acquisitions usually taking more than one hour [10].In this context, some PET tracers have been proposed as an effective alternative to mIBG. The most specific ones, which can assess the catecholaminergic NB pathway, can provide additional diagnostic information compared to mIBG scans and especially in high-risk (HR) NB patients, can change the clinical management [10]. PET/CT with catecholaminergic tracers are rarely used in clinical practice and not included in new therapeutic protocols for HR-NB patients, despite their apparent superiority and the recognition of their relevant diagnostic role by recent international procedural guidelines [4].

This evidence-based review aims to clarify the emerging diagnostic role of these PET tracers better by performing a systematic search of the literature to identify original studies reporting a head-to-head diagnostic comparison of mIBG to whole-body scan with PET/CT with catecholaminergic tracers such as meta-hydroxyephedrine ([^11^C]C-HED), ^18^F-18F-3,4-dihydroxyphenylalanine ([^18^F]DOPA) [^124^I]mIBG and *Meta*-[18F]fluorobenzylguanidine ([^18^F]mFBG).

## Materials and methods

The systematic review was conducted according to a predefined protocol and written according to the PRISMA statement [11].

### Search strategy

Two authors (A.P. and F.F.) searched the available literature independently. The search and selection process consisted of four separate steps.

In the first step, so-called “sentinel” studies were identified in PubMed by entering various combinations of the following keywords: ^11^C-HED, ^124^I-mIBG, ^18^F-mFBG, ^18^F-DOPA, PET/CT, mIBG, Neuroblastoma and PET. In the second step, the results were used to identify specific MeSH terms in PubMed. In the third step, PubMed, CENTRAL, Scopus, Web of Science and the web were searched using the selected MeSH terms. In the final step, we only included the studies that performed a head-to-head comparison among PET using catecholaminergic tracers and ^123^I-mIBG scintigraphy in identifying disease localisation in patients affected by NB. We excluded original articles without this head-to-head comparison. Review articles, studies based on preclinical data, phantom studies, case reports, and small case series (< 5 subjects) were also excluded.

The references of the included studies were searched to identify other potential matches. The search process was concluded on May 15^th^ 2023. Considering the heterogeneity of the studies, a meta-analysis was not planned or performed.

### Data extraction

The two authors (A.P. and F.F.) extracted independently:

1. General characteristics of the studies (authors, year of publication, country, study design, population).
2. Technical parameters (mode of acquisition, fasting before tracer injection and premedication, mean injected activity, uptake time, interval elapsed between the two imaging procedures, PET/CT scan field of view, PET/CT image analysis and use of reference standard).
3. Sensitivity of the two imaging procedures: this parameter was computed as a patient-based analysis (PBA) and a lesion-based analysis (LBA).
4. Standard of reference (SOR).

In the evaluation phase, full-text articles and their supplementary materials were included; in case of missing data, the responsible corresponding authors were contacted via e-mail. The extracted data were cross-checked, and any discrepancy was discussed through a consensus meeting.

The risk of bias in the studies was assessed by two authors (F.F and G.T.) by using the QUADAS-2 method [12]. For each study, an evaluation of the seven QUADAS-2 items was performed, and each point was scored as having a high, low or unclear risk of bias. High and unclear risk of bias were assigned 1 and 0.5 points, respectively; studies totalling a QUADAS-2 score of four or higher were excluded by the systematic review.

## Results

### Literature search outcome

**Fig. 1:**
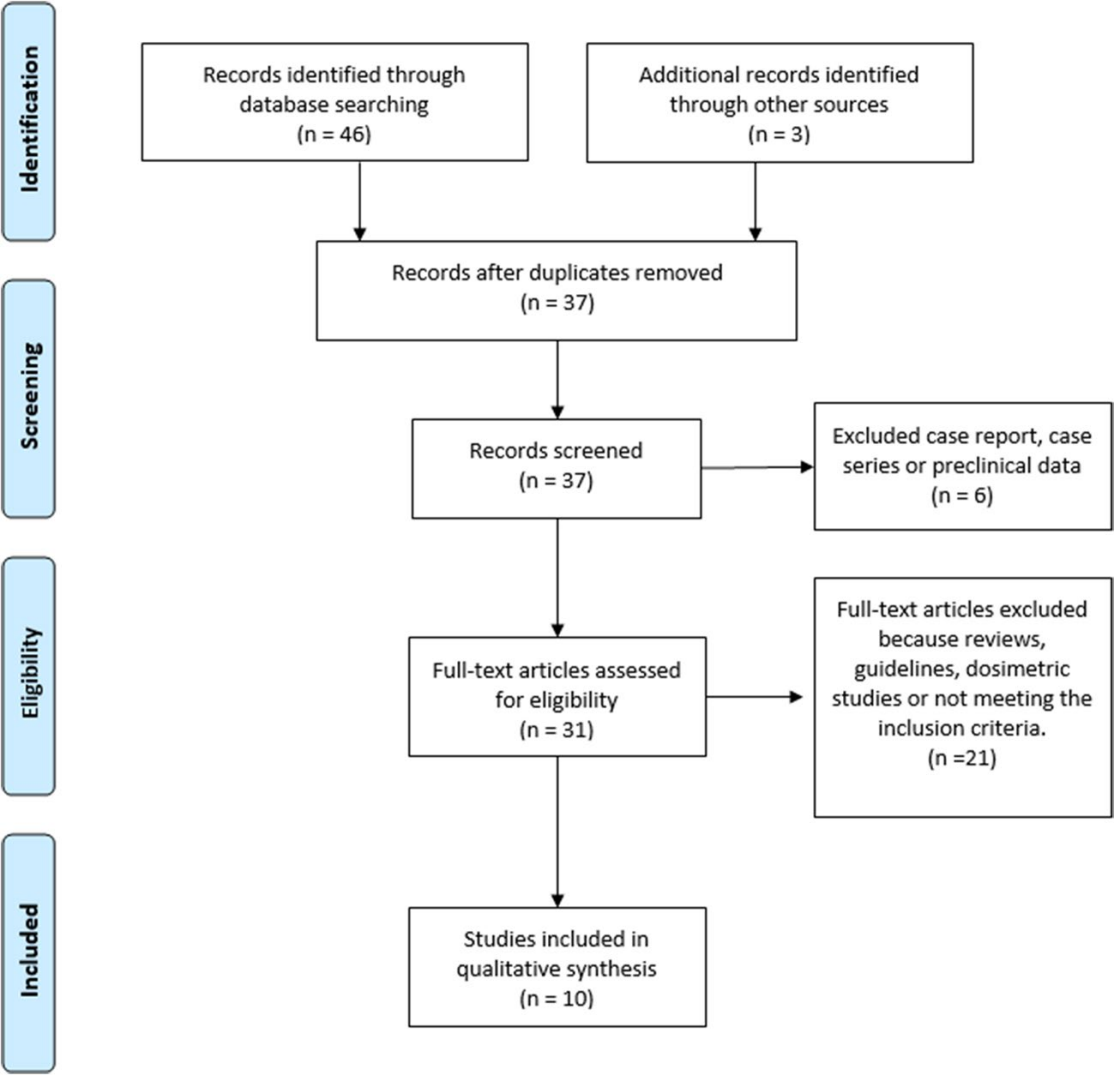
PRISMA flowchart indicating the selection process of the included studies

37 records were initially identified after duplicate removal, and their titles and abstracts were assessed; 6 articles had to be excluded since they reported on single cases, small cases series (< 5 subjects) or did not include human beings. Of the remaining 31 records, 21 were excluded because they did not meet the set inclusion criteria. Therefore, 10 articles were finally selected (Fig. 1).

### Qualitative analysis

The ten articles in the systematic review were published between 1996 and 2023 [13–22]. Five out of 10 had a prospective design [13, 16, 19, 21, 22]. Three studies were conducted in the USA and two others in Italy, while Germany, Taiwan, India, the Netherlands, and China each contributed one study. The characteristics of the studies and their patients’ populations are summarised in Table 1. Technical aspects are described in Table 2, diagnostic accuracy data are displayed in Table 3, and quality assessment of included studies is reported in Table 4.

**Table 1:** Study and patients’ characteristics

Authors	Year	Country	Study design	Patients*	PET tracer	Iodine isotope for mIBG	NB	Children/adolescents and adults	Staging/restaging	SOR
Shulkin et al. [14]	1996	USA	R	6	[^11^C]C-HED	[^123^I]	6 HR*	5/1	6/0	CT and MRI
Franzius et al. [15]	2006	Germany	R	7	[^11^C]C-HED	[^123^I]	3 HR*	6/1	3/4	Histology and CT
Piccardo et al. [16]	2012	Italy	P	19	[^18^F]F-DOPA	[^123^I]	17 HR*	17/2	4/15	Histology, CT and MRI
Lu et al. [20]	2013	Taiwan	R	18	[^18^F]F-DOPA	[^123^I]	N.R	18/0	N.R	Histology
Piccardo et al. [13]	2020	Italy	P	18	[^18^F]F-DOPA	[^123^I]	16 HR*	18/0	18/0	Histology, CT and MRI
Hemrom et al. [17]	2022	India	R	46	[^18^F]F-DOPA	[^131^I]	N.R	46/0	42/4	N.R
Aboian et al. [18]	2021	USA	R	8	[^124^I]mIBG	[^123^I]	8 HR*	N.R	0/8	cross-sectional imaging
Pandit-Taskar et al. [22]	2018	USA	P	5	[^18^F]mFBG	[^123^I]	N.R	N.R	0/5	N.R
Samim et al. [19]	2023	Netherlands	P	14	[^18^F]mFBG	[^123^I]	14 HR*	11/3	5/9	N.A
Wang et al. [21]	2023	China	P	40	[^18^F]mFBG	[^123^I]	33 HR*	NR	3/37	Histology, CT and MRI

^*^Included only those patients who performed both mIBG and PET with one of the catecholaminergic tracers

^**^HR High Risk Neuroblastomas

*NR* Not reported, *N/A* Not available

*R* retrospective, *P* prospective

**Table 2:** Technical aspects of PET and SPECT imaging in the included studies

Authors	mIBG	mIBG activity	SPECT imaging modality	Patients’ preparation before mIBG scan	mIBG uptake time	PET Tracer	Mean PET tracer activity	PET imaging modality	Patients’ preparation before PET	PET-tracer uptake time	Time between mIBG scan and PET	Image analysis	PET interpreted as positive when
Shulkin et al. [14]	[^123^I]mIBG	370 MBq	SPECT with PET-matching FOV	Thyroid gland blocking	24 h	[^11^C]HED	185 MBq	PET	NR	None	3–4 weeks	Visual analysis and TBR calculated	Uptake higher than surrounding background
Franzius et al. [15]	[^123^I]mIBG	EANM dosage card	SPECT/CT (primary tumour only)	Thyroid gland blocking	24 h	[^11^C]HED	320 MBq	PET/CT	NR	None	< 4 weeks	Visual analysis and SUV calculated	Uptake higher than surrounding background
Piccardo et al. [16]	[^123^I]mIBG	5.2 MBq/Kg	SPECT (15 patients)	Thyroid gland blocking (Lugol solution)	24 h	[^18^F]DOPA	4 MBq/Kg	PET/CT	Fasting state for at least 4 h	60 min	10 days	Visual analysis	Uptake higher than surrounding background
Lu et al. [20]	[^123^I]mIBG	7 MBq/Kg	SPECT (some patients, specific body parts)	Thyroid gland blocking (Lugol solution)	24 h	[^18^F]DOPA	4 MBq/Kg	PET/CT	2 mg/Kg of carbidopa orally 1 h before injection	90 min	concomitantly	Visual analysis, TBR and SUV calculated	NR
Piccardo et al. [13]	[^123^I]mIBG	EANM dosage card	SPECT/CT (thoraco-abdominal)	Thyroid gland blocking (Lugol solution)	24 h	[^18^F]DOPA	4 MBq/Kg	PET/CT	Fasting state for at least 4 h	60 min	10 days	Visual analysis and SUV calculated	Uptake higher than surrounding background
Hemrom et al. [17]	[^131^I]mIBG	25–49 MBq	SPECT/CT of the region-of-interest	Thyroid gland blocking (Lugol solution)	48-72 h	[^18^F]DOPA	5 MBq/Kg	PET/CT	NR	60 min	< 1 month	Visual analysis	Uptake higher than liver
Aboian et al. [18]	[^123^I]mIBG	5.2 MBq/Kg	SPECT/CT (focused FOV)	Thyroid gland blocking	24 h	[^124^I]mIBG	1.05 MBq/Kg	PET/CT	NR	24 h	< 2 weeks	Visual analysis and correlation with cross-sectional imaging	Uptake higher than surrounding background
Pandit-Taskar et al. [22]	[^123^I]mIBG	N.R	SPECT/CT of the chest, abdomen, and pelvis	Thyroid gland blocking	24 h	[^18^F]mFBG	148–444 MBq	PET/CT	N.R	60–120 min	< 4 weeks	Visual analysis	NR
Samim et al. [19]	[^123^I]mIBG	4 MBq/Kg	SPECT/CT of the area-of-interest	Thyroid gland blocking (Lugol solution, thiamazole, thyroxine)	24 h	[^18^F]mFBG	2 MBq/Kg	PET/CT	NR	60 min	< 2 weeks	Visual analysis	Uptake higher than surrounding background
Wang et al. [21]	[^123^I]mIBG	5.18 MBq/Kg	SPECT/CT (one or more bed positions)	Thyroid gland blocking	20-24 h	[^18^F]mFBG	2–3 MBq/Kg	PET/CT	NR	90 min	< 1 week	Visual analysis, TBR and SUV calculated	Uptake higher than surrounding background

*FOV* field-of-view, TBR* Target to Background Ratio, *NR* Not reported

**Table 3:** Data available in the *ten* studies included in the present systematic review

Authors	Year	PET Tracer	Patient-based analysis						Lesion-based analysis					
			Patients	mIBG scan +	Sen (%)	PET +	Sen (%)	p	Lesions	mIBG scan +	Sen (%)	PET +	Sen (%)	p
Shulkin et al. [14]	1996	[^11^C]HED	6	6	100	6	100	N.S	6	6	100	6	100	N.S
Franzius et al. [15]	2006	[^11^C]HED	7	7	100	7	100	N.S	33	32	97	32	97	N.S
Piccardo et al. [16]	2012	[^18^F]DOPA	19	11	68	16	95	0.01	136	80	59	118	87	< 0.01
Lu et al. [20]	2013	[^18^F]DOPA	18	12	75	16	100	0.045	18	12	67	16	89	N.S
Piccardo et al. [13]	2020	[^18^F]DOPA	18	17	94	18	100	N.S	578	514	89	564	97	< 0.01
Hemrom et al. [17]	2022	[^18^F]DOPA	46	39	85	42	91	N.S	369	229	62	363	98	< 0.01
Aboian et al. [18]	2021	[^124^I]mIBG	8	8	100	8	100	N.S	87	32	37	87	100	< 0.01
Pandit-Taskar et al. [22]	2018	[^18^F]mFBG	5	5	100	5	100	N.S	34	22	64	34	100	< 0.01
Samim et al. [19]	2023	[^18^F]mFBG	14	12	92	13	100	N.S	52^*^	18	35	52	100	< 0.001
Wang et al. [21]	2023	[^18^F]mFBG	40	30	89	34	100	N.S	784	532	67	784	100	< 0.001

*mIBG scan +* mIBG scintigraphy + SPECT or SPECT/CT true positive findings, *PET +* PET or PET/CT true positive findings, *Sen* sensitivity, *N.S.* non-significant, *NR* not reported

^*^including the primary tumours and the soft-tissue lesions; bone lesions have not been calculated since they were expressed through the SIOPEN score

**Table 4:** QUADAS-2 assessment of the included studies

		Risk of bias				Applicability		
First author	Year	Patient selection	Study test	Reference standard	Timing	Patient selection	Study test	Reference standard
Shulkin et al. [13]	1996	U	U	U	L	H	L	L
Franzius et al. [14]	2006	L	L	L	L	L	L	L
Piccardo et al. [15]	2012	L	U	U	L	L	L	L
Lu et al. [19]	2012	L	U	U	L	L	L	L
Piccardo et al. [10]	2020	L	L	L	L	L	L	L
Hemrom et al. [16]	2022	U	L	L	L	U	L	L
Aboian et al. [17]	2021	H	U	U	L	H	L	L
Pandit-Taskar et al. [21]	2018	U	U	L	L	L	L	L
Samim et al. [18]	2023	L	L	L	L	L	L	L
Wang et al. [20]	2023	L	L	L	L	L	L	U

*H* high, *L* low, *U* unclear

### Technical aspects

The scintigraphic imaging modality consisted of a whole body [^123^I]mIBG scintigraphy combined with SPECT in all studies (SPECT/CT in 7 studies). Acquisition started 24 h after the tracer injection. In only one out of ten studies, a whole-body [^131^I]mIBG scintigraphy with SPECT/CT was acquired 48-72 h after the tracer injection [17]. A proper thyroid-blocking protocol was scheduled in all the studies (Table 2).

PET/CT with low-dose CT was performed in all but one study, in which the CT component was not included[14]; four different catecholaminergic PET tracers were tested. [^11^C]C-HED and [^18^F]F-DOPA were used in two [14, 15] and four studies [13, 16, 17, 20], respectively, while [^124^I]mIBG and [^18^F]mFBG were employed in one [18] and three studies, respectively [18, 20, 21]. The fasting state was required only for [^18^F]F-DOPA. No other preparations were reported for the other PET tracers.

The injected activity ranged from 1 MBq/Kg for [^124^I]mIBG to 2 to 5 MBq/Kg for fluorinated PET tracers. For [^11^C]C-HED fixed doses of 185 and 320 MBq were employed (Table 2).

The time interval between radiotracer injection and PET image acquisition was similar across the studies using fluorinated PET tracer, being 60 min in 4 of these 7 studies [13, 16, 17, 19]. For [^11^C]C-HED, an early acquisition starting immediately after the tracer injection was employed [14, 15]. Finally, a 24 h acquisition was reported for [^124^I]mIBG [18].

In six studies, PET image analysis was performed by a combination of qualitative (visual) and semi-quantitative analysis through the calculation of the maximum standardised uptake values (SUV) or target-to-background ratio (TBR). In the remaining four studies, only a visual analysis was conducted (Table 2).

On visual analysis, a PET tracer uptake greater than the surrounding normal tissue that could not be explained by physiological activity was considered positive in eight studies. In the remaining two studies, the criteria for classifying PET findings as positive were not specified [19, 21]. All technical aspects are summarised in Table 2.

### Diagnostic performance

The ten articles selected for the systematic review were published between 1996 and 2023 and included populations consisting of 5 to 46 patients affected by Neuroblastic Tumours (Table 1). Overall, a head-to-head comparison between mIBG scintigraphy (combined with SPECT or SPECT/CT) and PET with catecholaminergic tracers is available for 181 patients (Table 2). Table 3 details the rate of positive cases at the PBA and LBA. It is important to emphasise that 97/112 (87%) patients were considered to have high-risk NB. Among the 80/163 NB (49%) patients for which the clinical setting was specified (i.e., staging or restaging), mIBG scintigraphy and PET was performed at the time of disease onset.

Overall, mIBG scintigraphy and PET with catecholaminergic tracers showed a good agreement and high sensitivity in detecting sites of disease of NB patients, especially at the time of first diagnosis [13, 14, 17]. This is particularly evident in the PBA (Table 3). Indeed, PET/CT with [^18^F]DOPA appears slightly more sensitive than scintigraphy with [^123^I]mIBG, with some studies reporting a statistically significant difference in favour of PET [16, 20].

**Fig. 2:**
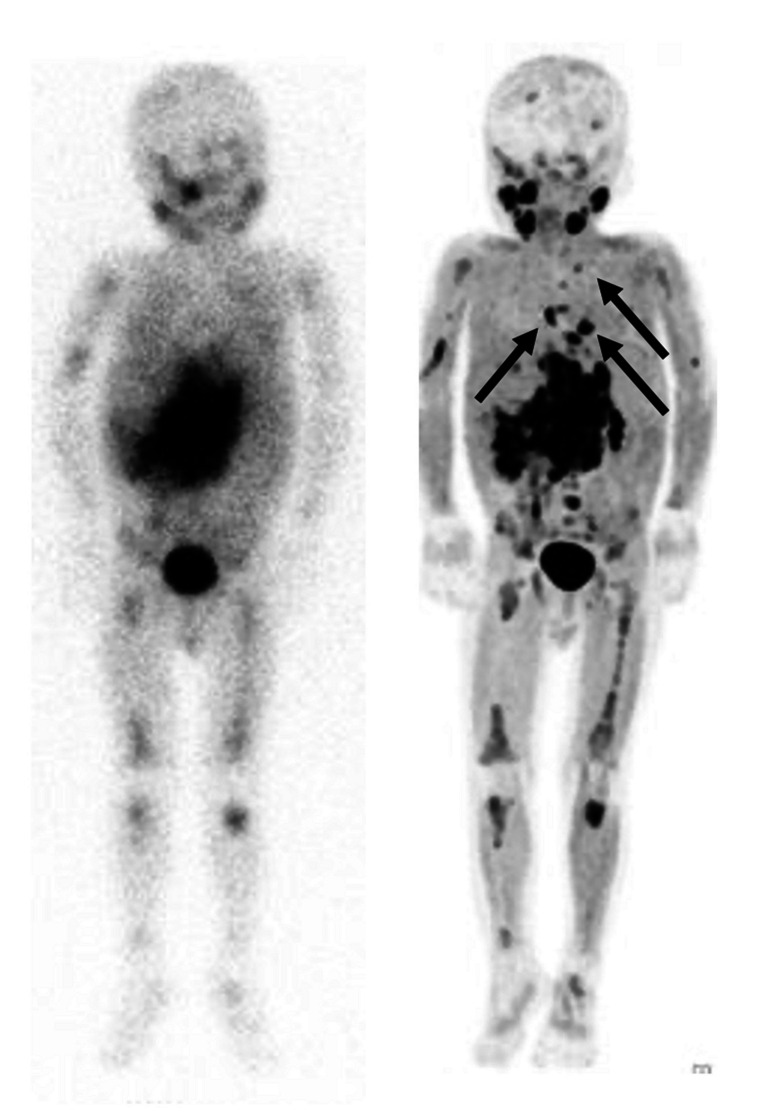
Therapy response evaluation of a 4-year-old boy with high-risk neuroblastoma. [^123^I]mIBG whole-body scan (left panel). Maximum intensity projection (MIP) of [.^18^F]mFBG PET (right panel). Pathological uptake in primary abdominal neuroblastoma and extensive osteomedullary neuroblastoma localisations on both investigations, more clearly and with higher resolution depicted with [18F]mFBG PET. [18F]mFBG PET also detected additional mediastinal lymph node metastases (arrows)

**Fig. 3:**
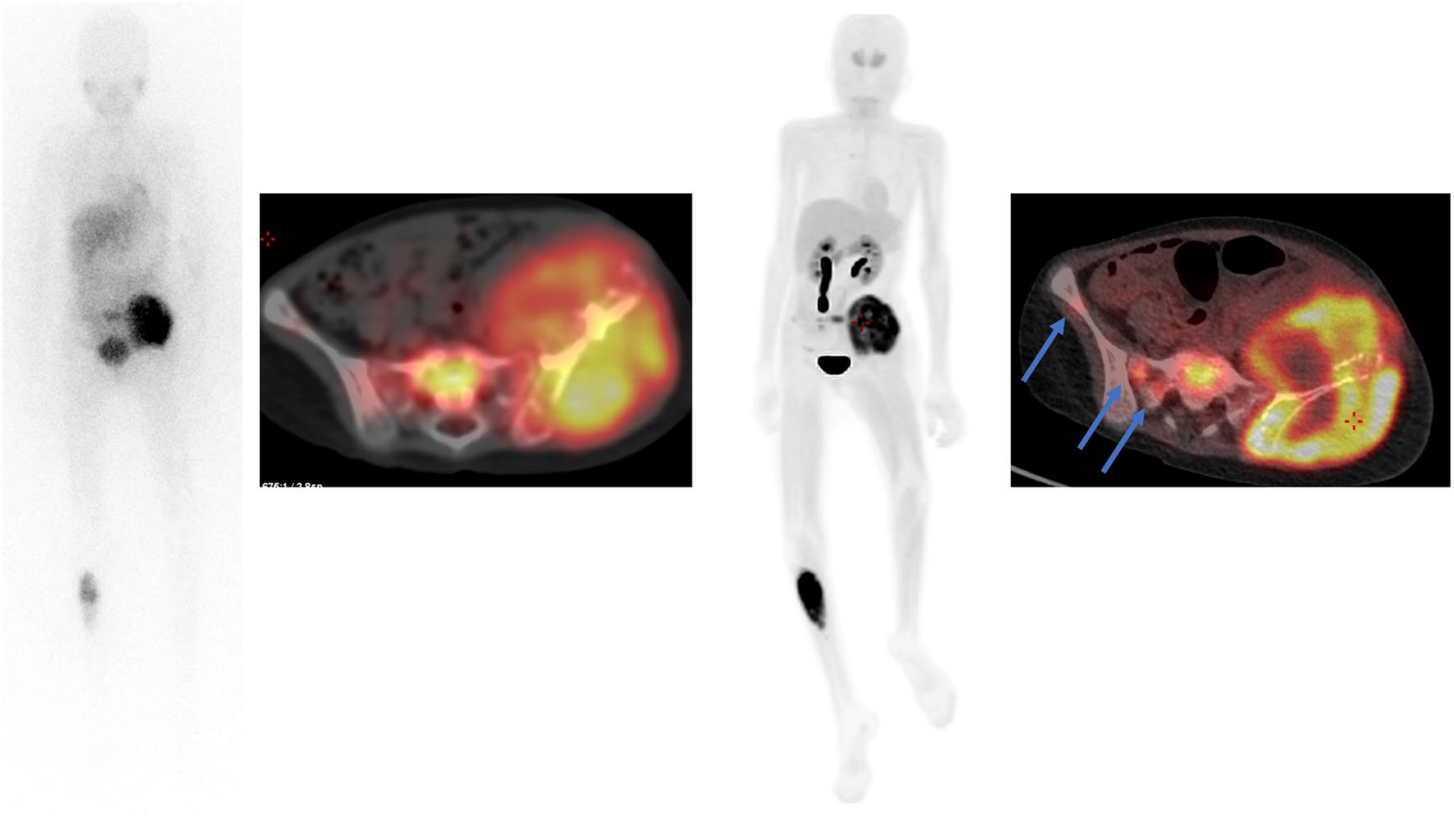
Sixteen-year-old boy affected by NB bone relapse. [^123^I] mIBG whole-body scan and SPECT/CT (planar and axial images) showed multiple bone localisations (left panels). In the same patients [^18^F]DOPA, maximum intensity projection (MIP) and axial images showed many small additional lesions (right panels, arrows)

On the other hand, when the ability to detect every single lesion was tested using a lesion-based analysis, PET imaging could disclose significantly more NB lesions than mIBG scintigraphy combined with SPECT or SPECT/CT in seven out of 10 studies (Table 3) [10, 15–18, 20, 21]. This difference was more evident in those studies that included patients with high-risk disease and with suspected or ascertained relapse. In this setting, when tumour load and dimensions are often not so conspicuous, PET imaging can identify even small lesions often not detected on an mIBG scan [18, 19, 23]. On the other hand, there were no significant differences in the LBA of studies employing [^11^C]C-HED [15]. Figures 2 and 3 show representative examples of the improved spatial resolution and sensitivity of PET methods over [^123^I]mIBG scan.

Regarding the different metastatic sites, PET imaging with catecholaminergic tracers revealed more soft tissue and bone-bone marrow metastases than [^123^I]mIBG scan [13, 16, 18, 19]. Additionally, it has been reported that PET/CT results can also influence patient management and/or the therapeutic strategy adopted based on all available data, including the initial mIBG scan report in about 30% of NB patients [16]. It must be pointed out that the setting in which the use of PET has the most potential to change the therapeutic strategy is disease relapse identification, where the identification of a single lesion by PET can make the difference between remission and disease recurrence. In contrast, identifying additional lesions in subjects with extensive disease spread is not likely to prompt any therapy modification.

### Quality assessment of the studies

The risk of bias was assessed according to seven items, which are listed in Table 4. The overall bias score ranged from none to 3; therefore, no study had to be excluded because of a high bias risk. The most frequent sources of possible bias were the “selection of patients” and “study test” since, in some studies, it was unclear whether a blinded evaluation of the two methods had been performed. In one case, an age-based selection was performed [18]. Risks of bias regarding feasibility were rarely detected.

## Discussion

In this systematic review, we aimed to clarify the differences in the diagnostic performance of catecholaminergic PET tracers and mIBG SPECT/CT in children with NB. More recently, some metabolic and receptor PET tracers, such as [^18^F]FDG or [^68^ Ga]-DOTA peptides[24], have been tested; however, even though they could be of complementary value when tumours show scarce mIBG uptake, their specificity is limited. Moreover, assessing disease extension or response to treatment by using one of these tracers as a single agent can be particularly difficult, especially in high-risk NB patients with predominant bone marrow involvement [25–27]. Currently, the most attractive and promising PET tracers remain those able to describe the catecholamine metabolic pathway, characterising with high specificity the neuroblastic tumours. In this study, we collected all the available evidence on the sensitivity of these tracers in patients affected by NB compared to mIBG scans. This is the first effort to analyse the evidence-based data of this special class of PET tracers in a non-negligible number of patients. Particularly, we evaluated the role of four catecholaminergic tracers such as [^18^F]DOPA, [^11^C]C-HED, [^124^I]mIBG and [^18^F]mFBG. Among these, one contains the same carrier molecule of the SPECT tracer (mIBG), adopting a different, positron-emitting isotope of the same element; mFBG also uses the same base structure, but it replaces iodine with fluoride. Finally, HED represents a catecholamine analogue, and DOPA is a precursor of dopamine and catecholamines [28].

Our qualitative assessment showed no significant differences in sensitivity between PET imaging and mIBG scan in the PBA, with both modalities providing similarly high sensitivity. However, the clinical setting and the selection of patients included in the analysis can explain the inconstant presence of significant differences between these imaging methods. Indeed, these patients were mostly affected by HR-NB and mainly evaluated at the time of first diagnosis when there is a high prevalence of true positive lesions [10, 13, 16]. Indeed, when we consider the studies including predominantly patients evaluated at restaging when highly sensitive procedures are required to identify even small persistent or relapsing lesions, a slight difference in favour of PET imaging was observed [15].

When we investigated the diagnostic sensitivity of these diagnostic tools using an LBA, we found that the sensitivity of PET with catecholaminergic tracers was reported to be significantly higher than that of mIBG-based tracers in 7 out of 10 studies. Particularly, the pathological distribution of the tracers seems to be very similar to that of mIBG; no mIBG-positive lesion was missed by [^18^F]- or [^124^I]-based PET. The higher ability of PET to disclose sites of disease was confirmed both for soft tissue and osseous/bone marrow metastases. This last finding has an influence on the Curie or SIOPEN scores which are based on the bone marrow disease extension. In addition, due to the higher diagnostic accuracy of catecholaminergic tracers, PET/CT can prompt modifications in the clinical management and therapeutic strategy in up to 32% of patients*.* However, the actual clinical impact of PET on clinical decision-making remains untested.

When analysing the sensitivity of the two methods, it must be pointed out that not all studies included SPECT/CT imaging as a part of the standard scintigraphy protocol. Moreover, even those who did employ SPECT/CT used somewhat inconsistent protocols, ranging from one-bed position to a PET-like field of view. This discrepancy could have theoretically affected the comparison between [^123^I]mIBG. On the other hand, it must be considered that three-dimensional hybrid imaging is more likely to improve the sensitivity of the method in specific bodily districts, which can normally be encompassed in a single SPECT/CT bed position. Consequently, even though the sensitivity of the scintigraphic method on LBA showed significant variation, this variability did not appear to be linked to the acquisition protocol (SPECT vs one-bed SPECT/CT vs “PET-like” SPECT/CT)*.*

All this growing body of evidence indicates that these PET imaging procedures are effective and reliable in detecting NB localisation. In addition, their accuracy is significantly higher than that of mIBG scans. This important diagnostic advantage over mIBG is accompanied by numerous practical advantages. [^123^I]mIBG scanning is a time-consuming procedure, requiring at least one acquisition to be performed the next day after the tracer injection [10]. In paediatric hospitals not equipped with nuclear medicine capabilities, the patient must be transferred at least twice to an external facility. Achieving adequate image quality requires a long scanning time (up to one hour), which decreases patients’ comfort and compliance; moreover, in smaller children, extended periods of sedation may be required [4]. Planar images are often difficult to interpret, with high tracer background contribution, and decisions with relevant therapeutic impact must sometimes be made based on the presence of faint areas of uptake. The advent of SPECT/CT has significantly improved the accuracy of lesion detection; however, this technique comes at the cost of yet another acquisition time increase and whole-body SPECT/CT is rarely achieved [29, 30]. Moreover, validated interpretation scores are not yet calibrated to account for the information provided by SPECT/CT [30].

We believe the limitations described with [^123^I]mIBG imaging can be mitigated using PET/CT with appropriate neuroblastoma tracers. PET images can be acquired a mere hour after tracer injection; they have superior resolution with little background noise and include the anatomical information for the entirety of the field of view, with a scanning duration of 15–20 min [10]. Newer devices feature a long axial field-of-view detector set, allowing scans to be made in as little as two minutes or using one-tenth of the activity required for regular tomographs [31]. With a long axial-field-of-view device, whole-body imaging will allow true quantification of the tracer distribution, as well as kinetics analyses; advanced image analysis, such as texture analysis and potentially machine learning algorithms, to be developed [32]. Considering these data, PET/CT appears to be superior in all aspects compared to [^123^I]mIBG scanning; moreover, when using tracers labelled with [^18^F]- or [^124^I], it appears to identify all of, and occasionally more than, the lesions identified on [^123^I]-mIBG planar and SPECT imaging.

It has to be pointed out, however, that not all PET radiopharmaceuticals are equal. [^11^C]HED is one of the oldest ones and bears the disadvantages linked to [^11^C] labelling, namely short physical half-life (requiring an on-site cyclotron) and a relatively poor PET resolution[33]. Accordingly, this tracer did not perform better than [^123^I]MIBG in the head-to-head [15]. As such, it does not represent a promising [^123^I]mIBG alternative. [^124^I]mIBG is, on the other hand, a theragnostic tracer, sharing the same molecular structure as [^123^I]mIBG, and its long half-life (4.2 days) allows for dosimetry application [18, 34]. On the other hand, such a long decay time implies a higher radiation exposure for the children; moreover, [^124^I] has a very complex decay scheme, with only roughly 20% of the emitted radiation being high-energy positrons. These characteristics can degrade the image quality [35]. [^18^F]F-DOPA represents the most studied PET tracer in children with NB, making up more than half of the patients in the current review. It is an FDA/EMA-approved radiopharmaceutical which can be used in many clinical settings, even beyond oncology; its use in NB is supported by the most current diagnostic guidelines [4]. Even though it does not represent a theragnostic tracer, there is evidence that its distribution mirrors the one of mIBG [36]. Finally, [^18^F]mFBG also uses the same base structure as mIBG and is labelled with [^18^F], the most commonly used PET isotope is the most promising alternative to [^123^I]mIBG but is not yet widely available [19, 37].

Managing children with HR-NB is a complex endeavour, requiring multiple stages of disease assessment and an accurate therapy response evaluation. Treatment of metastatic NB relies heavily on molecular imaging, which can track the presence of residual clonal burden and identify the disease relapse that can have relevant therapeutic consequences. Children facing this ailment are often infants and almost ever scared of undergoing imaging; their discomfort is paralleled only by that of their parents or caregivers. In this setting, having a two-day or a one-hour procedure can make an enormous difference in their well-being; the shorter alternative could be associated with greater patients’ compliance, leading to better image quality. Moreover, the ease of interpretation provided by PET fosters smoother and more confident decision-making in the context of the nuclear medicine physician evaluation and the multidisciplinary board meeting. Finally, the accumulating evidence on the advantages of PET in NB[38] should promote its use in research studies as well; however, current trials still rely on single-photon catecholaminergic imaging [5]. Considering all these points, it is clear that efforts should be made to promote PET imaging in HR-NB, both in day-to-day use and in clinical research. Such a transition would bring about better imaging, better patient care, and a higher chance of improving the current treatment standards through focused research.

This systematic review has some limitations, such as the low number of patients and articles included. The relatively low number of patients is coherent with the disease prevalence. The number of articles included is explained by the strict inclusion criteria used in our systematic review (only studies comparing [^123^I]mIBG scans with PET using catecholaminergic tracers were included); moreover, the comparison between [^123^I]mIBG with PET tracers was always of direct nature (i.e., based on a head-to-head analysis rather than on two separate comparisons with the gold standard). Moreover, half of the studies were prospective investigations, limiting the possible selection and information bias strongly. In almost all cases, the proof of truth consisted of the follow-up imaging, while a histological confirmation was only rarely available; this limitation stems from the impossibility of obtaining a biopsy from each disease localisation in patients with diffuse metastatic disease and cannot be easily circumvented in such analyses. In one case, PET imaging was compared with [^131^I]mIBG [17]; this radiopharmaceutical has an inferior spatial resolution as compared with [^123^I]mIBG (due to the higher energy of the gamma photons emitted by [^131^I]I) and a longer uptake time. Moreover, the dose burden of [^131^I]mIBG is higher than [^123^I]mIBG, thus limiting the activity that can be safely administered and reducing the resulting counting rate. These characteristics could impact the sensitivity of the method. On the other hand, [^131^I]mIBG is significantly cheaper than [^123^I]mIBG and could represent an accessible diagnostic resource in low-income or emerging countries in the coming years*.* Also, the included data allowed calculation of the sensitivity of the methods but not their specificity. The SPECT or SPECT/CT field-of-view did not match the one of PET/CT in all cases, being limited to a single bed position in some studies. However, since most patients were children, even such a field of view could likely encompass most patients’ thoracic and abdominal regions. Furthermore, the superiority of the PET-based tracers on LBA appears to be independent of the disease localisations (axial skeleton vs. the appendicular segments). As shown in the tables, heterogeneous findings among the included studies should be recognised. For this reason, we did not perform a pooled analysis (meta-analysis) of diagnostic performance.

## Conclusions

Catecholaminergic PET tracers appear to provide a more accurate evaluation of the disease burden compared to standard single-photon gamma camera imaging; this improvement is likely to stem from the higher resolution of the method. This increase in sensitivity can have a potentially relevant impact on the therapeutic decisions made by treating clinicians; however, the real clinical impact of PET in this setting remains to be proved. Moreover, PET offers many advantages, such as faster scanning time, single-day protocol, and no need for thyroid blockade, that can be invaluable in smaller children undergoing multiple catecholaminergic examinations. The increased accuracy, the quantification capability and the possibility to perform advanced image analysis could make PET imaging a superior choice for neuroblastoma clinical and research protocols.

## Data Availability

All data used in drafting the current manuscript can be retrieved online from their respective sources.
